# Hydrophilic Molecularly Imprinted Chitosan Based on Deep Eutectic Solvents for the Enrichment of Gallic Acid in Red Ginseng Tea

**DOI:** 10.3390/polym11091434

**Published:** 2019-09-01

**Authors:** Guizhen Li, Kyung Ho Rwo

**Affiliations:** Department of Chemistry and Chemical Engineering, Inha University, Incheon 402751, Korea

**Keywords:** hydrophilic molecularly imprinted chitosan, deep eutectic solvents, solid phase microextraction, gallic acid, response surface methodology

## Abstract

Hydrophilic molecularly imprinted chitosan (HMICS) were synthesized based on hydrophilic deep eutectic solvents (DESs) and the DESs was used as both a template and functional monomer for the enrichment of gallic acid (GA) from red ginseng tea using a solid phase microextraction (SPME) method. Using the response surface methodology (RSM) strategy, the optimal extraction amount (8.57 mg·g^−1^) was found to be an extraction time of 30 min, a solid to liquid ratio of 20 mg·mL^−1^, and five adsorption/desorption cycles. Compared to traditional methods, the produced HMICS-SPME exhibited the advantages of simplicity of operation, higher recovery and selectivity, improved analytical characteristics and reduced sample and reagent consumption, and it is expected to promote the rapid development and wide applications of molecular imprinting.

## 1. Introduction

Gallic acid (GA) is one of the major polyphenolic compounds in plants, such as green tea, vegetables and fruits [1,2]. GA is used in the pharmaceutical and biomedical industries owing to their anti-oxidant, anti-inflammatory, anti-cancer, and anti-viral activities [3,4].

Chitosan (CS) is the deacetylated form of chitin and has been used widely in material-formulations because of its distinct advantages, such as biocompatible, biodegradable, less toxic, and bioactive chemistry properties [5,6,7]. Moreover, CS is an eco-friendly and cost-effective biopolymer that can be modified easily by various chemical reactions to improve its physicochemical properties [8]. CS can be modified mainly using its amine group, by crosslinking reactions to make it insoluble in acidic pH or by grafting new functional groups to the amine and hydroxyl groups to add new chemical properties and improve the selectivity for the targets [9]. With the addition of new functional groups on CS, it is possible to increase the number of adsorption sites [10].

Currently, the molecular imprinting technique has attracted increasing attention for the production of artificial materials with selective recognition for the target molecules [11]. Molecular imprinting is an approach of artificially generating recognition sites in polymer structures to specifically rebind the target molecules. These materials are obtained by polymerizing functional and cross-linking monomers around a template molecule, leading to a highly cross-linked three-dimensional network polymer. With different kinds of imprinting methods, many products of the molecular imprinting technique have been shown with excellent selectivity and unique structural predictability, such as molecularly imprinted polymers (MIPs) [12], molecular imprinted resins (MIRs) [13,14], and molecular imprinted nanoparticles (MINs) [15]. Practical molecular imprinting materials (MIMs) have become a rapidly evolving research area, providing key factors for understanding the separation, recognition, and regenerative properties toward biological molecules [16].

For further applications, however, MIMs should have good water compatibility because many targeted molecules are present in the aqueous biological matrix [17]. Therefore, imprinting materials need to be modified for use in the aqueous phase system. In molecular imprinting techniques, deep eutectic solvents (DESs) has been used as the solvent for template elution and additive in the preparation of imprinting materials for higher adsorption capacity, acting as monomers [18], solvents [19], or others [20]. DESs are a eutectic mixture of a quaternary ammonium salt as a hydrogen bond acceptor (HBA) with either the organic amine, alcohol, or organic acid as the hydrogen bond donor (HBD) [21], which are characterized by a melting point lower than those of each individual component. Recently, some efforts have been made to introduce DESs in the fields of molecular imprinting [22] and for the separation of bioactive compounds because of their unique properties, including low vapor pressure, easy preparation, and benign biodegradability [23].

In this study, a novel hydrophilic molecularly imprinted chitosan (HMICS) was synthesized using DESs for the selective enrichment of GA in red ginseng tea leaves via a solid phase microextraction (SPME) method. The DESs were used as both the template and functional monomer. The materials obtained were characterized by scanning electron microscopy (SEM), Fourier transform infrared spectroscopy (FT-IR), thermogravimetric analysis (TGA), and nuclear magnetic resonance spectroscopy (NMR). The adsorption kinetics and isotherms for the adsorption models were also investigated. The optimal extraction conditions for GA were optimized using a response surface methodology (RSM).

## 2. Experimental

### 2.1. Chemical and Reagents

Red ginseng tea was purchased from a local market (Incheon, Korea). Methanol, GA, acetic acid, sodium hydroxide, protocatechuic acid, 3-hydroxybenzoic acid, 4-hydroxybenzoic acid, protocatechuic acid, and 3,5-dihydroxybenzoic acid were supplied by Sigma-Aldrich. Co, Ltd. (St Louis, MO, United Stated). CS powder (molecular weight (*M*_w_), 75 kDa; degree of deacetylation (DD), 80 mol %) was obtained from Seafresh Chitosan (Lab, Incheon, Korea) Co. Acetonitrile, acetone, hexane, ethyl acetate, liquid paraffin, span-80, glutaraldehyde, isopropanol, choline chloride, and azobisisobutyronitrile (AIBN) were acquired from Daejung Chemicals & Metals Co., Ltd. (Gyonggido, Yongin, Korea). The other chemicals used were of high performance liquid chromatography (HPLC) grade.

### 2.2. Instrument

The surface morphology was examined by field emission SEM (Hitachi S-4200, Hitachi, Toronto, ON, Canada). FT-IR (Vertex 80 V, Bruker, Billerica, MA, USA) spectroscopy was conducted to examine the functional groups between 4000 and 400 cm^−1^ using KBr pellet samples. The formation of particles was confirmed by TGA (TG 209 F3, Netzsch, Selb, Germany) under a nitrogen atmosphere from room temperature to 800 °C at a heating rate of 10 °C·min^−1^. The chromatographic measurements were performed on a high performance liquid chromatography (HPLC) system using YL9110 equipment from Younglin Co. Ltd. (Daegu, Korea) consisting of a Rheodyne injector (20 μL sample loop), Waters 600 s Multi solvent Delivery System, Waters 1515 liquid chromatography (Waters, Bedford, MA, USA), and variable wavelength 2489 UV dual channel detector. EmpowerTM 3 software (Waters, Bedford, MA, USA) was used as the data acquisition system. The analysis was performed on an OptimaPak C_18_ column (5 μm, 250.0 mm × 4.6 mm, i.d., RStech Corporation, Daejeon, Korea). All the 1H NMR experiments were carried out with a Bruker DMX 300 spectrometer (Bruker, Karlsruhe, Germany), equipped with a diffusion probe capable of producing magnetic field gradient pulses up to 11.76 T/m in the z-direction. The measurements were carried out in a temperature range between 293.8 and 300 K. A Bruker Variable Temperature unit, BVT 3000 (Bruker, Karlsruhe, Germany), was used to set the required temperature for each experiment. The sample was placed in a 5 mm NMR glass tube with a height of approximately 20 mm and left for 20 min at the desired temperature in order to reach thermal equilibrium.

### 2.3. Preparation of the Materials

#### 2.3.1. Synthesis of Hydrophilic DESs

Three types of hydrophilic DESs were prepared by mixing choline chloride with GA (1:1, *n/n*, DES-1), (1:2, *n/n*, DES-2), (1:3, *n/n*, DES-3) heated under 80 °C with constant stirring. After 8 h, the resulting DESs were obtained until a homogeneous colorless liquid had formed. Table S1 lists the basic physicochemical properties of the obtained hydrophilic DES. Only DES-2 (1:2, *n/n*) had a stable form prepared and was used in the following procedure.

#### 2.3.2. Preparation of HMICS and NICS

**Figure 1:**
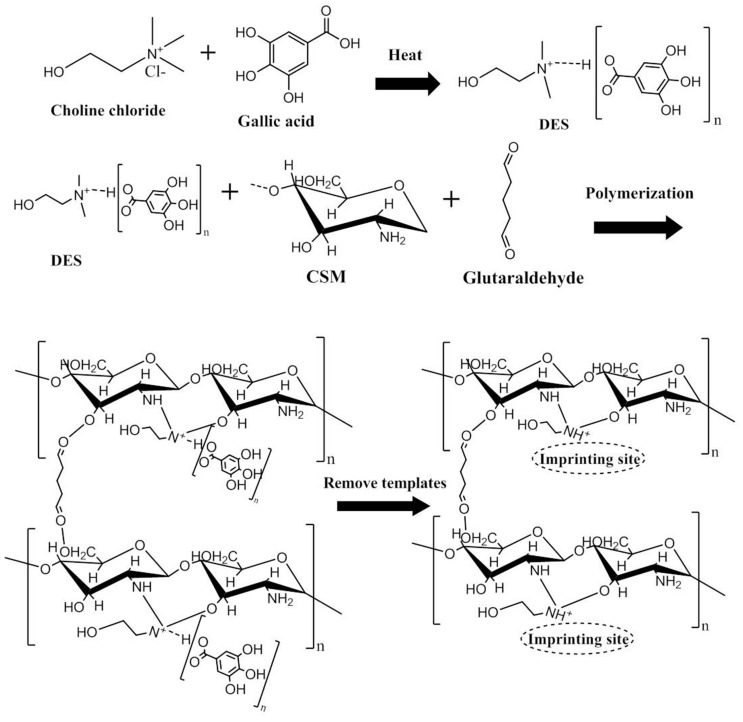
Schematic diagram of the preparation of hydrophilic molecularly imprinted chitosan (HMICS) with deep eutectic solvents (DES).

CS microspheres were obtained using an emulsion polymerization technique considering the requirements of high adsorptive surface areas and controllable preparation route [24]. A 10.0 g sample of CS was dissolved in 50.0 mL of a 5.0% (*w/v*) acetic acid solution and 25.0 mL isopropanol with constant stirring. A 2.0 mL sample of the above CS liquid mixture was then dispersed as the aqueous phase into 2.0 mL of liquid paraffin containing 10% (*v/v*) Span 80. Subsequently, 2.0 mL of the obtained hydrophilic DES-2 (both as a template and monomer) was added followed by the dropwise addition of (5%, 1.0 mL) glutaraldehyde solution (as a crosslinker). Following this, 0.5 g of AIBN was added as an initiator, and the homogeneous mixture was stirred at 60 °C for 3 h. The reacting mixture was left to stand at room temperature overnight for complete polymerization. The obtained materials were washed sequentially with petroleum ether and deionized water, and dried in a vacuum oven at 60 °C until a constant weight was reached. Figure 1 shows a schematic diagram of the preparation of HMICS with DES. Non-imprinted chitosan (NICS) was obtained in an identical manner, except that the template was not added during the preparing process of HIMCS, and Figure S1 shows the NMR spectroscopy of HMICS (a) and NICS (b).

#### 2.3.3. Adsorption Properties of HMICS and NICS

Static and dynamic adsorption experiments were conducted to evaluate the adsorption properties of the obtained HMICS and NICS. The adsorption capability for HMICS and NICS was determined by the following procedure and model. Briefly, 20 mg of HMICS and NICS were suspended in 10 mL of a GA solution at concentrations ranging from 5 to 200 μg·mL^−1^. The series of mixtures were shaken for 240 min under 25 °C to ensure the equilibrium.

The adsorption capacity (Q) was calculated according to the following equation:

(1) $$Q=\frac{\left(C_{0}-C_{\mathrm{free}}\right)\times V}{W}$$

where C_free_ (μg·mL^−1^) is the free concentration of GA; C_0_ (μg·mL^−1^) is the initial concentration; V (mL) is the volume of the GA solution; and W (mg) is the mass of the materials.

For the adsorption kinetics study, 20 mg of HMICS and NICS was suspended in 10 mL of a 100 μg·mL^−1^ GA solution and shaken at 25 °C. The concentrations of GA from 30 to 360 min at a certain interval (30 min) were centrifuged and calculated using the following equation:

(2) $$\ln\left(Q_{e}-Q_{t}\right)=\ln(Q_{e})-k_{1}t$$

where Qt (mg·g^−1^) and Qe (mg·g^−1^) are the amount adsorbed at the given time and equilibrium, respectively; k_1_ (min^−1^) is the rate constant of the adsorption.

#### 2.3.4. Selectivity and Reusability Experiments

To estimate the selectivity of the obtained imprinted materials, selectivity experiments were conducted on GA along with protocatechuic acid, 3,5-dihydroxybenzoic acid, 3-hydroxybenzoic acid, and 4-hydroxybenzoic acid as competitive compounds. A 2 mg sample of HMICS or NICS was added to 1 mL of the mixture solution containing 100 μg/mL of the above five compounds. After shaking for 4 h, the solutions were collected by centrifugation and analyzed by HPLC. 

The imprinting factor (α) and selectivity factor (β) were used to evaluate the properties of selectivity of HMICS and NICS toward the template molecule (GA) and analogs (protocatechuic acid, 3,5-dihydroxybenzoic acid, 3-hydroxybenzoic acid, and 4-hydroxybenzoic acid). The α and β were calculated from the following equations:

(3) $$\alpha =\frac{Q_{\mathrm{HMICS}}}{Q_{\mathrm{NICS}}}$$

(4) $$\beta =\frac{\alpha_{\mathrm{template}}}{\alpha_{\mathrm{analog}}}$$

where Q_HMICS_ and Q_NICS_, and α_template_ and α_analog_ are the sorption capacity and imprinting factor toward GA (template) or the analog on the HMICS and NICS, respectively.

To test the regeneration capability, a 2 mg sample of HMICS or NICS mixed in the 1 mL of GA standard solution were evaluated by ten sequential cycles of adsorption-regeneration.

#### 2.3.5. Optimization of the SPME Conditions

**Figure 2:**
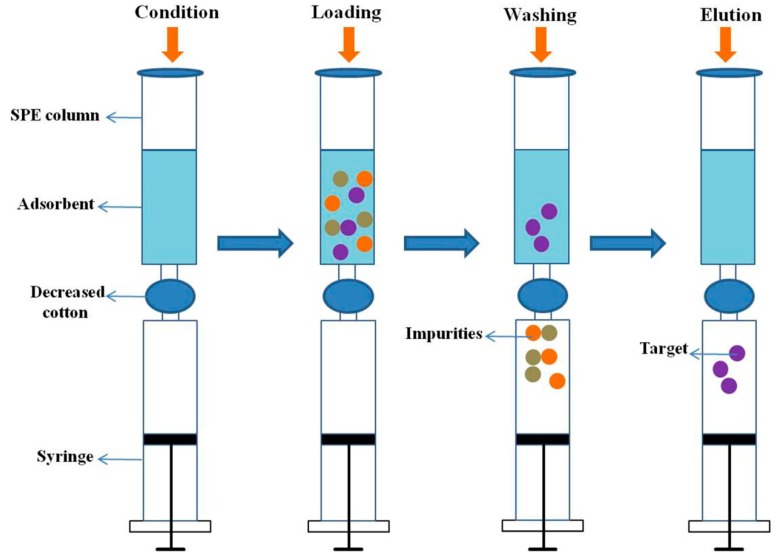
Schematic illustration of the miniature solid phase microextraction (SPME) procedure.

Red ginseng tea was cleaned, dried in an oven at 60 °C, and ground to a powder. A 10 g sample dried powder was ultrasonicated in 200 mL MeOH/water (80:20, *v/v*) at room temperature for 6 h. The suspension was then filtered as the extraction samples. The miniature SPME procedure was performed using SPE unit. A 20.0 mg sample of the obtained adsorbents was packed into SPE cartridges and connected to a conventional syringe to ensure a suitable and constant flow rate, and capped with decreased cotton in the middle of the SPE cartridges and the syringe. Figure 2 presents a schematic diagram of the miniature SPME procedure.

To remove interferents from matrix, different washing solvents, including methanol, acetonitrile, acetone, hexane, and ethyl acetate solutions, with different volumes (0.2–2.0 mL) were tested. The washing solvent was forced to pass through the system by regulating the vacuum of approximately 20 kPa to obtain a flow rate of 0.5 mL·min^−1^.

To select the most appropriate eluent to desorb GA from the prepared HMICS, several eluent solvents (methanol, methanol-acetic acid (85:15, *v/v*), methanol-ammonia water (85:15, *v/v*), acetonitrile-acetic acid (85:15, *v/v*), and acetone-acetic acid (85:15, *v/v*)) were tested. The washing solvent was forced to pass through the system by regulating the vacuum of approximately 20 kPa to obtain a flow rate of 0.5 mL·min^−1^.

#### 2.3.6. SPME Procedure with Real Samples Using the RSM

The RSM was applied to determine the optimal levels of the three variables having a significant effect on the extraction efficiency. After determining the preliminary range of the analysis variables through a single-factor test, the experimental variables were designed to optimize the adsorption efficiency of GA. The effects of the three independent variables, namely extraction time (X_1_, min), solid to liquid ratio (X_2_, mg·mL^−1^), and number of adsorption/desorption cycles (X_3_) on the extraction yields of analytes were investigated using a Box-Behnken design (BBD) of 17 experimental points.

Each variable coded at its three levels (−1, 0, 1) represents the lower, middle and higher value (Table S2). The generalized second-order polynomial Equation (5) used in response surface analysis is as follows:

(5) $$Y=A_{0}+\overset{3}{\underset{i=1}{\sum }}A_{i}X_{i}+\overset{3}{\underset{i=1}{\sum }}A_{\mathrm{ii}}X_{i}^{2}+\overset{2}{\underset{i=1}{\sum }}\overset{3}{\underset{j=i+1}{\sum }}A_{\mathrm{ij}}X_{i}X_{j}$$

where Y is the measured response; A_0_ is a constant; A_i_, A_ii_, and A_ij_ are linear, quadratic, and interaction coefficients, respectively; and X_i_ and X_j_ are the levels being studied. Data analysis was performed using Design-Expert software (v.7.1.6, Stat-Ease, Inc., Minneapolis, MN, USA) and evaluated by an analysis of variance (ANOVA). The fitness of the polynomial equation to the responses was estimated using the coefficient of determination (*R*^2^), and the differences with a p-value less than 0.05 were considered significant.

## 3. Results and Discussion

### 3.1. Adsorption Properties

**Figure 3:**
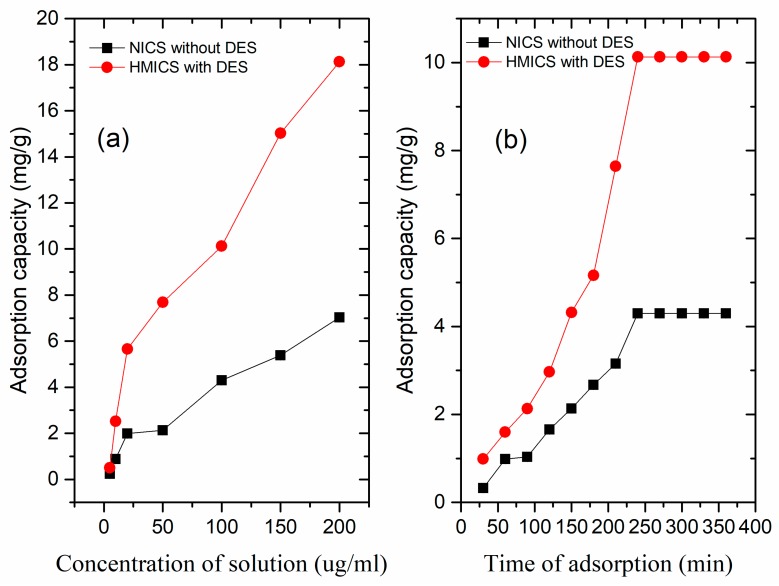
Equilibrium adsorption isotherms (**a**) and kinetic adsorption curves (**b**) of HMICS with DES, and non-imprinted chitosan (INCS) without DES.

The binding capacity of HMICS and NICS increased with increasing GA concentration, and the adsorption capacity of HMICS with DES-2 was much higher than NICS without DES (Figure 3a). The additional GA bound to HMICS compared to NICS could be attributed to the binding of GA to the imprinting sites with higher specificity. 

The adsorption kinetics of GA onto HMICS and NICS was investigated by varying the adsorption time from 30 to 360 min (Figure 3b). The adsorption capacity increased rapidly in the first 0–100 min, and then the increment from 125–225 min until the process approximately reached equilibrium after 225 min. At the beginning of the adsorption process, the GA could enter many empty specific binding sites easily and rapidly and mass-transfer resistance was significantly small. With time prolonging, it became difficult to find an imprinted site for target. Therefore, the adsorption rate decelerated up to reaching equilibrium. A sharp increase in the adsorption amounts towards GA by HMICS and NICS occurred within 240 min. The adsorption capacity of GA on HMICS with DES was 10.13 mg·g^−1^, which is approximately 2.36 times as high as that (4.30 mg·g^−1^) of NICS without DES. The fast and greater dynamics of HMICS adsorption were attributable to the imprinted sites.

### 3.2. Characterization

**Figure 4:**
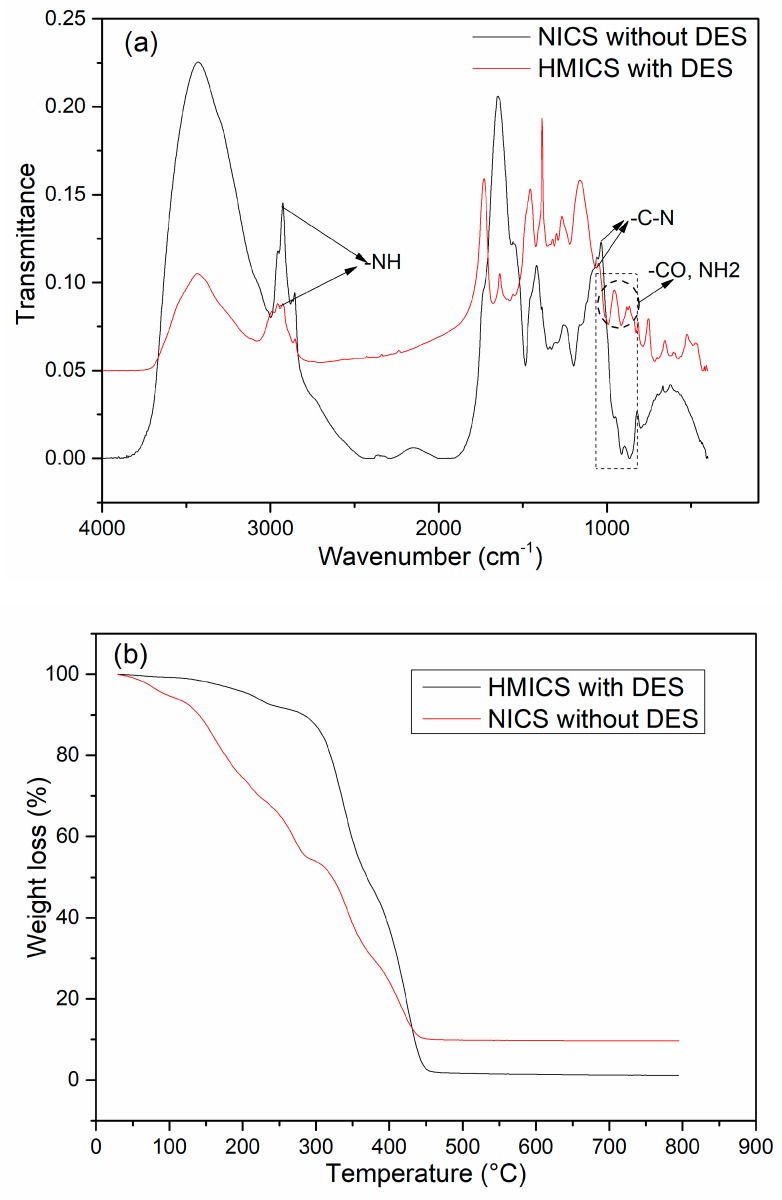
FT-IR spectra (**a**) and thermogravimetric analysis (**b**) of HMICS with DES, and NICS without DES.

Figure 4a presents the FT-IR spectra of HMICS and NICS. The absorbance at 3400−3500 cm^−1^ was assigned to the overlapping stretching vibration of the O-H bonds and N–H bonds on the NICS surface. The absorption at approximately 1620 cm^−1^ in HMICS was attributed to the bending vibration of NH_2_, which provides evidence of the presence of DES in the HMICS skeleton. In addition, a characteristic absorption band at approximately 1154 cm^−1^ is related to a C−N stretching vibration. The absorbances at 2900 and 1650 cm^−1^ were assigned to the overlapped stretching vibration of CH groups and CO groups, respectively. These results demonstrate the successful formation of HMICS.

TGA was conducted to estimate the thermal stability of the prepared HMICS and NICS, as shown in Figure 4b. HMICS showed 9.2% weight loss between 25 and 300 °C, which was attributed to the elimination of free water and structural water molecules, whereas NICS showed a small mass loss due to the evaporation of residual water from 0 °C and 50 °C. When the temperature was increased to more than 200 °C, the organic shell gradually lost weight and decomposed completely at 400 °C. TGA of HMICS showed significant mass loss from 300 °C to 450 °C, whereas NICS without DES showed significant mass loss from 50 to 420 °C. These weight losses were attributed to the decomposition and vaporization of a grafted macromolecular microsphere. The weight loss of both of HMICS and NICS remained relatively constant from 450 °C to 800 °C. Therefore, HMICS had good thermal stability below 300 °C, whereas NICS was stable below 50 °C.

**Figure 5:**
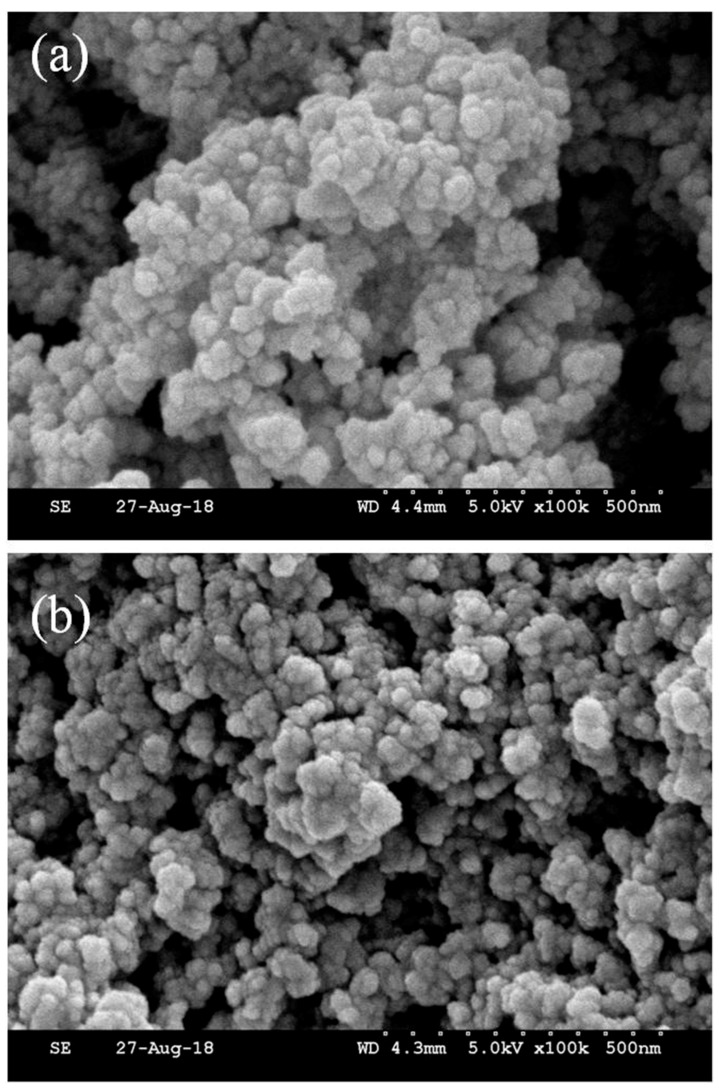
SEM images of HMICS with DES, (**a**), and NICS without DES (**b**).

SEM images were obtained to observe the shape and morphology of HMICS and NICS; the images revealed a uniform structure with a spherical morphology. As shown in Figure 5, HMICS had a more uniform structure with a more regular spherical morphology than NICS. Moreover, the HMICS possessed a slightly spherical structure with a relatively greater size distribution, which facilitated mass transfer and rapid sorption kinetics.

### 3.3. Optimization of the Extraction Conditions 

**Figure 6:**
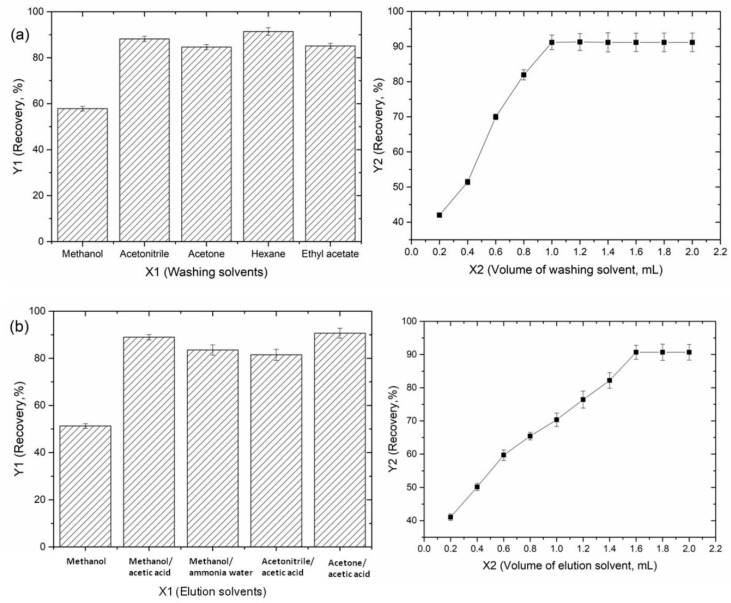
Optimization of the HMICS in the SPME procedure for GA ((**a**): Washing solvents, (**b**): Elution solvents).

Owing to the complex of the tea sample matrices, it is essential for further validation and optimization of the washing and elution conditions. A washing step was required to remove the other constituents of the tea samples bound nonspecifically to the imprinted sorbent. Regarding the chemical structure of the GA, solubility of the target analyte and its compatibility with chromatographic system, the washing solvents (acetonitrile/acetone/hexane/ethylacetate), showed efficient, which may be due to the similarity between the polarity of the target and the washing solvents could break hydrogen bonds between trapped analyte and sorbent easily. The cleanest extract with the highest recovery was obtained using hexane as the washing solvent. Different hexane volumes (0.2–2.0 mL) were tested and the optimal value (91.4%) was 1.0 mL (Figure 6a). Because of the hydrophilicity of HMICS and high potential of hexane to dissolve non-polar compounds, most of the matrix interfaces were washed with hexane without interfering with the interactions between the GA and sorbent.

The selection of a suitable eluent solute has a great influence on the extraction recovery of a target molecule. According to the experimental results, the best recovery was achieved using acetone-acetic acid (85:15, *v/v*) as the eluent (Figure 6b). Different eluent volumes (0.2–2.0 mL) were tested and a volume of 1.6 mL of acetone-acetic acid (85:15, *v/v*) was found to be sufficient to desorb the analyte. The addition of acetic acid could impede hydrogen bonding between GA and the stationary phase, leading to the easier removal of GA.

### 3.4. Selectivity and Reusability of HMICS and NICS

**Figure 7:**
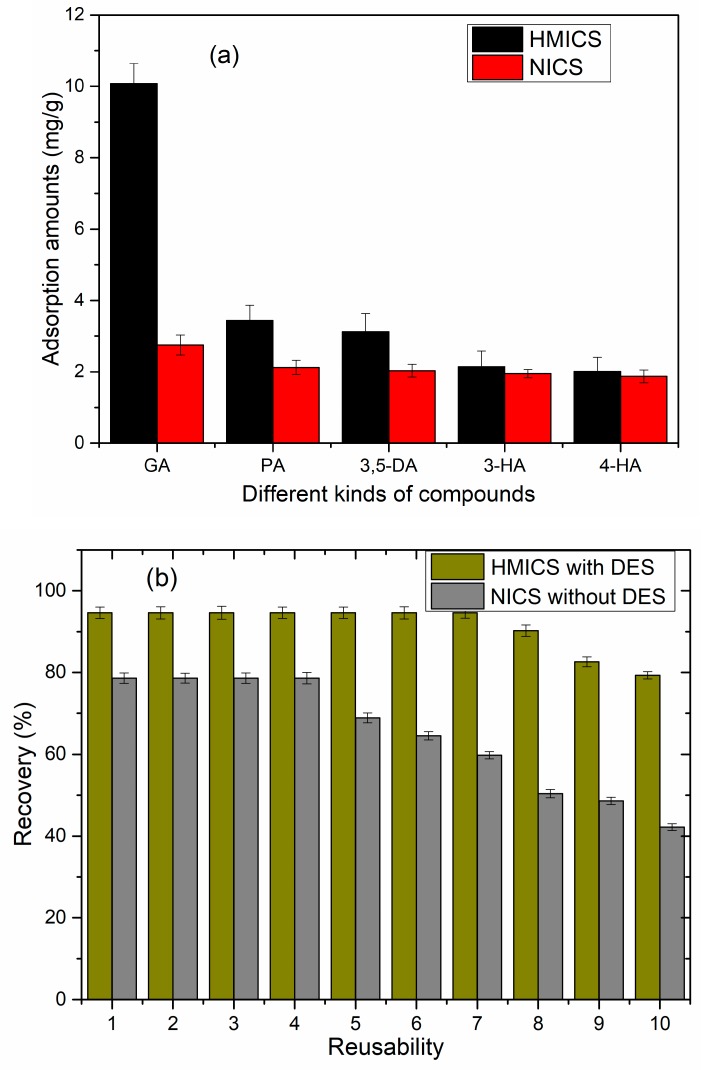
Selectivity (**a**) and reusability (**b**) of HMICS with DES, and INCS without DES. (GA: Gallic acid; PA: Protocatechuic acid; 3,5-DA: 3,5-Dihydroxybenzoic acid. 3-HA: 3-Hydroxybenzoic acid; 4-HA: 4-Hydroxybenzoic acid)

Imprinting factor and selectivity factor are the chief and prominent advantages of imprinted materials. The imprinting and selectivity capability of HMICS were evaluated by comparing the imprinting factor (α) and selectivity (β) of GA in the presence of competitive compounds. As shown in Figure 7a, α values of HMICS for GA, protocatechuic acid, 3,5-dihydroxybenzoic acid, 3-hydroxybenzoic acid, and 4-hydroxybenzoic acid were 3.67, 1.62, 1.53, 1.09, and 1.07, respectively, and the β values of HMICS for GA, protocatechuic acid, 3,5-dihydroxybenzoic acid, 3-hydroxybenzoic acid, and 4-hydroxybenzoic acid were 0.70, 0.22, 0.20, 0.14, and 0.13, respectively. It was shown that the HMICS had the highest selectivity value toward GA, and it was further verified that GA was adsorbed onto the HMICS by means of specified imprinted sites. Moreover, these results show that HMICS had highly selective recognition capability toward GA.

Reproducibility and reusability are very important for designing an advanced and effectual sorbent. As shown in Figure 7b, the extraction efficiency of the HMICS remained at a relatively high level, even after seven cycles, whereas NICS showed an obvious decrease at the fourth cycle, indicating that the HMICS sorbent can be employed frequently as an effective sorbent for GA recovery.

### 3.5. RSM Model Fitting and Statistical Analysis

The extraction variables optimized for the extraction efficiency for GA were the extraction time, solid to liquid ratio, and number of adsorption/desorption cycles. Table S3 lists the central composite experimental design with the independent variables. The quadratic response surface regression model was used to predict the extraction efficiency in terms of the extraction parameters (coded factors), as expressed in Equation (6):

*Y* = 92.92 + 0.29*X*_1_ − 1.34*X*_2_ + 1.72*X*_3_ + 0.025*X*_1_*X*_2_ + 0.75*X*_1_*X*_3_ + 0.25*X*_2_*X*_3_ − 0.70*X*_1_^2^ + 1.00*X*_2_^2^ − 4.42*X*_3_^2^ (6)

Table S4 lists the ANOVA results for the quadratic response surface regression model and the significance of the regression coefficients to maximize the extraction recovery. The F-value of the model was 13.47 and the p-value was less than 0.0001, indicating that the model was significant in predicting the extraction yield. The Model *F*-value of 13.47 suggested that the model was not significant relative to the noise, and there was only a 0.12% probability that a “Model F-Value” could occur due to noise. The “Lack of Fit F-value” of 5.46 suggested that the Lack of Fit was not significant relative to the pure error, and there was a 6.74 % chance that a “Lack of Fit F-value” could occur due to noise. Moreover, its corresponding p-value was 0.0674, indicating that the model fitted the experimental data well.

The coefficient of determination (*R*^2^ = 0.9454) indicated that 94.54% of the variability in the extraction recovery could be explained by this model (Table S5). Moreover, the predicted extraction recovery values were close to those from the BBD, which confirmed the reliability of the model. The difference between the predicted *R^*2*^_Pred_* (0.2814) and adjusted *R^*2*^_Adj_* (0.8752) indicates there is reasonable agreement in the regression polynomial model. The signal to noise ratio was measured using “Adeq. Precision”; a ratio of greater than four was normally desirable. The “Adeq. Precision” of 11.623 suggested that this model could be used to navigate the design space.

**Figure 8:**
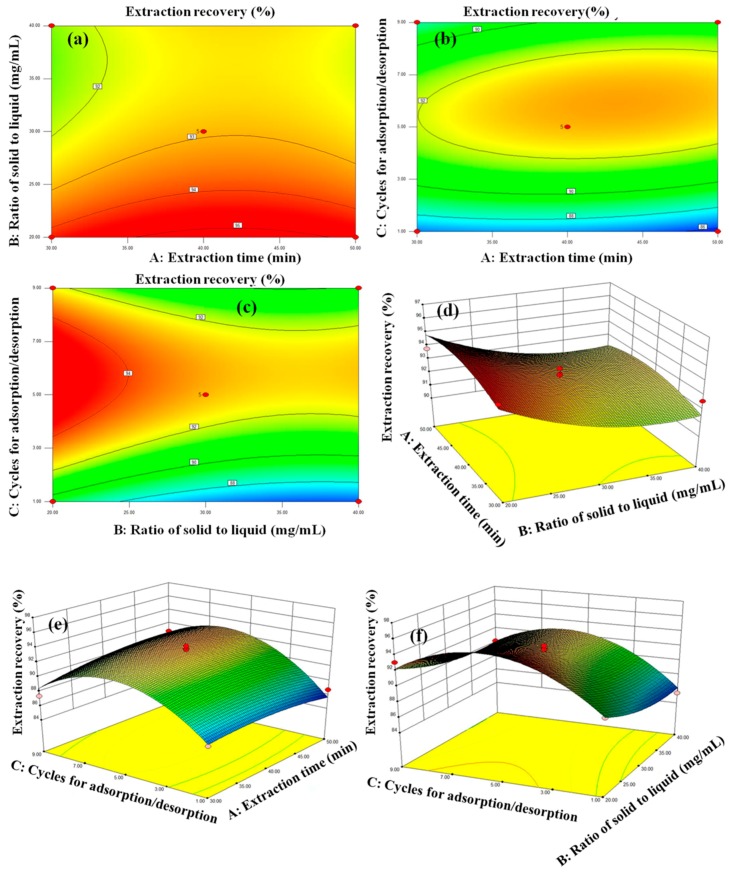
The contour plot (**a**–**c**), and reciprocal 3D response interaction (**d**–**f**) on the HMICS extraction recovery yield of three variables (effects of extraction time, ratio of solid to liquid, cycles for adsorption/desorption).

The contour plot and three-dimensional (3D) response surfaces were plotted to investigate the interactions among the variables (Figure 8). The extraction yield decreased with increasing extraction time ranged from 30 min to 50 min, increased with increasing solid to liquid ratio from 20 mg·mL^−1^ to 30 mg·mL^−1^, and then decreased from 30 mg·mL^−1^ to 40 mg·mL^−1^. Regarding the number of adsorption/desorption cycles, in the designed ranges of one to nine, the extraction recovery increased and then decreased. The theoretical maximum extraction recovery (94.6%) for GA was obtained at an extraction time of 30 min, solid to liquid ratio of 20 mg·mL^−1^, and five adsorption/desorption cycles. Under the optimal extraction conditions, the actual extraction recovery was 93.9%, which is close to 94.6%, highlighting the suitability and accuracy of the suggested models.

### 3.6. Method Validation and Real Sample Analysis

The standard curve for GA was linear over the range, 5.00–100.00 μg·mL^−1^, by assaying five data points in triplicate (Y = 3.78 × 10^4^ + 2.54 × 10^4^X, R^2^ = 0.9997). To validate the developed method for the determination of GA, further experiments with regard to the calibration linearity range, limit of detection (LOD), limit of quantification (LOQ), recovery, and relative standard deviation (RSD) were conducted under the optimized experimental conditions (Table 1). The LOD (0.32–0.41 μg·mL^−1^) and LOQ (0.22–0.36 μg·mL^−1^) were calculated as three and ten times the standard deviation of the noise signal, respectively. The recoveries of the targets extracted did not differ significantly and the RSD was no higher than 4.16%, demonstrating the high selectivity of the proposed method.

**Figure 9:**
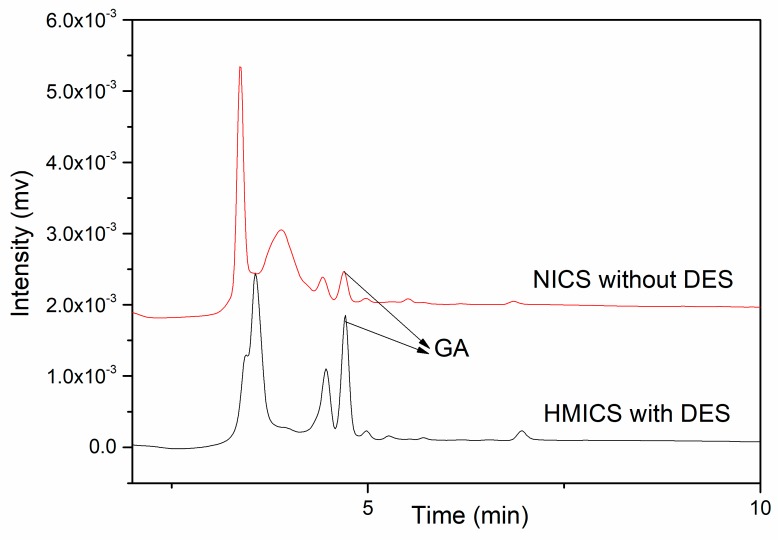
Extraction chromatograms of red ginseng tea extracts with HMICS with DES, and INCS without DES. (Column: C_18_ column, mobile phase: (Acetonitrile-0.05% phosphoric acid solution = 5:95, *v/v*), flow rate: 1.0 mL·min^−1^, UV: 270 nm, injection: 20 μL).

As shown in Figure 9, the red ginseng tea extract is a complex matrix and there were some peaks other than that for GA. After pretreatment with HMICS, the interfering peaks weakened. A significant peak for GA was observed, confirming that GA in the tea extract sample could be extracted selectively by HMICS, and the GA content in red ginseng tea was 8.57 mg·g^−1^.

### 3.7. Comparison with other Methods

To highlight the distinct merits of the HMICS and present method, a comparison with other reported studies was made, as shown in Table 2. The obtained HMICS had lower LODs than previous imprinted materials for the extraction of GA.

The developed method provided a wide linear range and a much lower LOD than the other methods, whereas the recovery and precision of this method were comparable to or better than the other methods. Therefore, the developed HMICS-SPME method can be used as an effective method for the simple, rapid, cost-effective, sensitive, and selective determination of GA in red ginseng tea samples.

## 4. Conclusions

A novel HMICS was synthesized based on a hydrophilic DES used as both the template and functional monomer for the enrichment of GA from red ginseng tea using the SPME method. The optimal extraction amount (8.57 mg·g^−1^) was found at an extraction time of 30 min, solid to liquid ratio of 20 mg·mL^−1^, and five adsorption/desorption cycles using the RSM strategy. Compared to the traditional CS microspheres, the HMICS produced using the hydrophilic DESs exhibited higher extraction capacity. Such improvements will allow an extension of the field of application of the new imprinted CS, which could become a new tool used routinely in analytical laboratories in the future.

## Figures and Tables

**Table 1:** Intra-day and Inter-day precisions and accuracy of GA.

Targets	Concentration (μg·mL^−1^)			Inter-day		Intra-day	
		Limit of Detection (μg·mL^−1^)	Limit of Quantification (μg·mL^−1^)	Recovery (%)	Relative Standard Deviation (%, n = 4)	Recovery (%)	Relative Standard Deviation (%, n=4)
Gallic Acid	50	0.21	0.24	90.13	4.16	90.04	3.68
	100	0.15	0.18	94.82	3.84	93.68	3.15
	200	0.08	0.13	102.68	2.65	100.84	3.06

**Table 2:** Comparison of different materials for extraction of GA.

Materials Types	Function Monomer	Source	Recovery (%)	Reference
Molecularly imprinted microparticles	Methacrylic acid	Olive mill wastewaters	85.0–97.0	[25]
Molecularly imprinted microspheres and nanoparticles	Acrylic acid	Emblica officinalis	75.0–83.4	[26]
Hydrophilic molecularly imprinted chitosan microsphere	DES	Tea sample	90.0–102.7	This research

## Supplementary Materials

The following are available online at https://www.mdpi.com/2073-4360/11/9/1434/s1, Table S1 Density (ρ), viscosity (μ), and conductivity (σ) of the three kinds of DESs at 293.15 K and atmospheric pressure (1.01 bar); Table S2 Independent variables their levels used for BBD; Table S3 Central composite experimental design with the independent variables; Table S4 Analysis of variance of the experimental results of the BBD; Table S5 Analysis of variance for the fitted quadratic polynomial model of extraction of GA; Figure S1. 1H NMR spectra of the HMICS (a) and NICS (b).
