# Supplementary material for: Hydrophilic Molecularly Imprinted Chitosan Based on Deep Eutectic Solvents for the Enrichment of Gallic Acid in Red Ginseng Tea
Source: Polymers (Basel). 2019 Sep 1;11(9):1434. doi: 10.3390/polym11091434 (PMC6780181; doi:10.3390/polym11091434)
Supplement: Supplementary file 1 [file polymers-11-01434-s001.pdf]

Supplementary Materials: Hydrophilic Molecularly Imprinted Chitosan Based on Deep Eutectic Solvents for the Enrichment of Gallic Acid in Red Ginseng Tea

Guizhen Li and Kyung Ho Rwo

Table S1 Density (ϱ), viscosity (μ), and conductivity (σ) of the three kinds of DESs at 293.15 K and atmospheric pressure (1.01 bar).

Abbreviations	Molar ratios (HBA: HBD)	Density ($g{\cdot}cm^{-3}$)	Viscosity (Pa·S)	Conductivity ($uS{\cdot}cm^{-1}$)
DES-1	1:1	1.1432	0.1896	1205.6
DES-2	1:2	1.1601	0.2042	1187.4
DES-3	1:3	1.1865	0.2146	1158.7

Table S2 Independent variables their levels used for BBD.

Variables	Level		
	-1	0	1
Extraction time (min) (X_1)	30	40	50
Ratio of solid to liquid ($mg{\cdot}mL^{-1}$) (X_2)	20	30	40
Cycles for adsorption/desorption (X_3)	1	5	9

Table S3 Central composite experimental design with the independent variables.

Run	Extraction recovery (%)				
	X_1	X_2	X_3	Actual values	Predicted values
1	-1	0	-1	86.3	86.0
2	0	0	0	93.2	92.4
3	-1	1	0	92.6	91.1
4	1	-1	0	93.8	94.3
5	0	1	1	90.2	89.6
6	0	-1	1	93.1	91.8
7	1	0	1	90.8	90.1
8	-1	-1	0	94.6	93.8
9	1	0	-1	86.7	85.1
10	0	-1	-1	89.3	88.9
11	0	0	0	93.6	92.4
12	0	1	-1	85.4	85.5
13	1	1	0	91.9	91.7
14	0	0	0	92.0	92.4
15	0	0	0	92.7	92.4
16	-1	0	1	87.4	87.9
17	0	0	0	92.2	92.4

Table S4 Analysis of variance of the experimental results of the BBD.

Variables	Sum of Squares	Diversfication Factors	Mean Square	F Value	p value Probability>F
Model	129.47	9	14.39	13.47	＜0.0001
X_1	0.66	1	0.66	0.62	0.4572
X_2	14.31	1	14.31	13.40	0.0081
X_3	23.80	1	23.80	22.29	0.0022
$X_1 X_2$	2.500E-003	1	2.500E-003	2.341E-003	0.9628
$X_1 X_3$	2.25	1	2.25	2.11	0.1899
X_2X_3	0.25	1	0.25	0.23	0.6433
X_1^2	2.05	1	2.05	1.92	0.2086
X_2^2	4.23	1	4.23	3.96	0.0868
X_3^2	82.35	1	82.35	77.11	＜0.0001
Residual	7.48	7	1.07	-	-
Lack of fit	6.01	3	2.00	5.46	0.0674
Pure error	1.47	4	0.37	-	-
Correlation total	136.94	16	-	-	-

Table S5 Analysis of variance for the fitted quadratic polynomial model of extraction of GA.

Item	Standard deviation	Mean	Coefficient variation %	Press	Coefficient	Adjusted coefficient	Predicted cofficient	Adequate precision
Value	1.03	90.98	1.14	98.41	0.9454	0.8752	0.2814	11.623

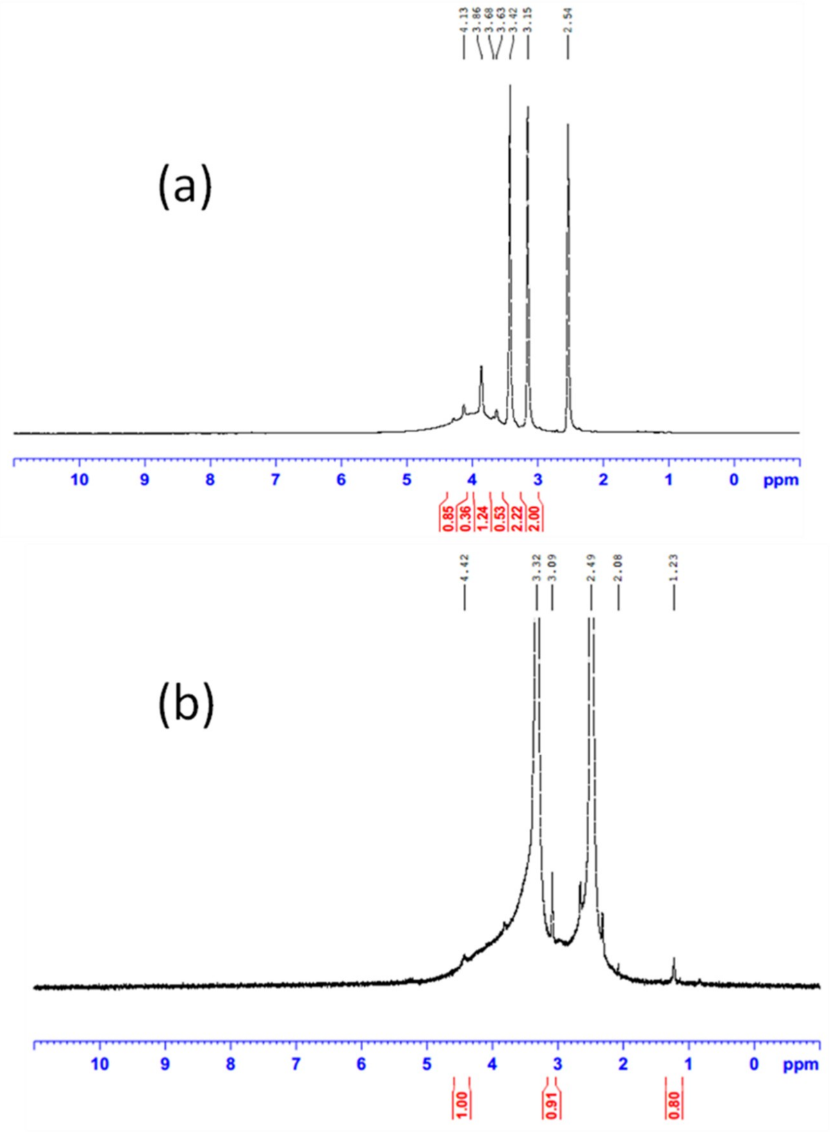

Figure S1 ^{1}H NMR spectra of the HMICS (a) and NICS (b).
